# Application of blood flow restriction training in adolescents: A narrative review

**DOI:** 10.1097/MD.0000000000043084

**Published:** 2025-07-18

**Authors:** Zhen-Lei Chen, Tian-Shu Zhao, Shuang-Feng Ren, Si-Zhuo Zhang, Ji-Lai Xu, You-Qing Shen, Li-Ping Huang

**Affiliations:** a School of Physical Education, Hubei University of Education, Wuhan, Hubei Province, China; b College of Sports Medicine and Rehabilitation, Beijing Sports University, Beijing, China; c College of Physical Education, International Equestrian school, Wuhan Business University, Wuhan, Hubei Province, China; d Graduate School of Medicine, Juntendo University, Tokyo, Japan.

**Keywords:** adolescents, blood flow restriction training, low-load exercise, physical health, strength training

## Abstract

Blood flow restriction training (BFRT) involves applying external compression to the limbs to restrict venous blood return during low-intensity exercise, thereby promoting improvements in muscle mass and strength. Originally developed decades ago, BFRT has gained renewed interest in recent years, with applications spanning rehabilitation medicine, aerospace, and general fitness. However, its use in adolescents remains limited and under-researched. This narrative review aims to summarize the current understanding of the effects, mechanisms, and practical applications of BFRT in adolescents, with a focus on muscle health, physical performance, and training safety. A comprehensive review of recent literature was conducted, focusing on BFRT-related physiological mechanisms, including metabolic stress, anabolic hormone responses, muscle fiber recruitment, protein synthesis regulation, myostatin suppression, and cell swelling. Relevant studies on adolescent populations were analyzed to evaluate the efficacy and safety of BFRT in this age group. BFRT appears to promote muscle hypertrophy, strength, endurance, and neuromuscular adaptations in adolescents, with reduced injury risk compared to high-load training. Individualized arterial occlusion pressure settings enhance both the safety and effectiveness of BFRT. Applications span general fitness, athletic performance enhancement, and injury rehabilitation. BFRT offers a promising, low-risk alternative to traditional resistance training for adolescents, supporting safe and effective physical development. Its integration into youth fitness and rehabilitation programs by educators, healthcare providers, and sports organizations may offer substantial benefits for adolescent health and performance.

## 1. Introduction

A recent survey in China highlighted that, during the COVID-19 pandemic, young adolescents experienced increased screen time and inadequate physical activity due to prolonged periods at home. More than half of Chinese youth temporarily adopted an unhealthy lifestyle marked by sedentary behaviors and poor physical activity levels.^[1]^ This physical inactivity poses risks to the growth, development, and mental health of adolescents (defined as individuals aged 10–19 years according to the World Health Organization, inclusive of both sexes), reinforcing the need for safe and effective physical exercise options specifically suited to this age group. However, traditional high-load strength training often presents challenges for adolescents due to their unique physiological needs, limited neuromuscular control, and increased susceptibility to injury from high-impact or high-resistance exercises. Research indicates that traditional training methods can sometimes lead to improper movement patterns and heightened injury risks, potentially limiting their effectiveness in improving overall physical fitness.^[2]^

Blood flow restriction training (BFRT) has emerged as a potentially safer alternative that may better meet adolescents’ needs by providing low-load resistance with high fitness benefits.^[3]^ BFRT involves applying external compression to the limbs to restrict venous blood flow, creating a hypoxic environment that stimulates muscle hypertrophy and strength gain, which are typically observed with high-load training.^[4]^ Importantly, this low-load characteristics of BFRT may reduce the risk of training-related injuries in adolescents, making it a potentially more suitable option for younger populations. Studies have suggested that BFRT may offer a higher benefit-to-risk ratio compared to traditional strength training due to its lower injury risk and comparable strength gains at reduced intensities.^[4]^

Initially developed as “KAATSU training” (a term now associated with a specific company and trademarked equipment), BFRT is a technique integrated into existing exercise protocols rather than a standalone exercise method.^[5]^ It is often applied with low-load interval training, leveraging the hypoxic environment to induce muscle adaptation similar to high-load exercises.^[6]^ BFRT has been widely studied in competitive sports and medical rehabilitation, showing effectiveness in improving muscle strength, endurance, and cardiovascular fitness in adults and aiding rehabilitation across musculoskeletal, neurological, and cardiovascular domains.^[5,7,8]^ However, research on BFRT’s safety and efficacy specifically in adolescents – particularly nonathlete – remain limited. Given adolescents’ unique growth and developmental considerations, it is essential to explore BFRT’s specific mechanisms and applications for this age group.

This study aimed to address this gap by investigating BFRT’s potential benefits and mechanisms in adolescents. We explore BFRT’s mechanisms of action and its practical applications for improving adolescent fitness, aiming to provide theoretical support and guidance for its implementation in this population.

## 2. The mechanism of BFRT in adolescents

**Figure 1.**
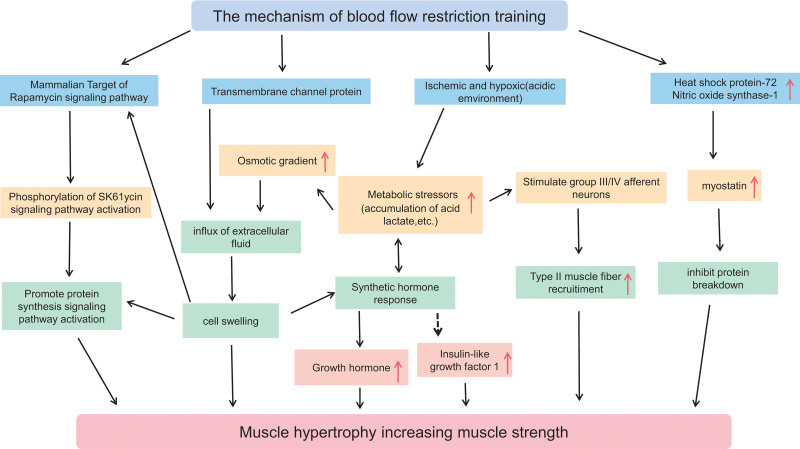
Mechanisms of BFRT in promoting muscle hypertrophy and strength increase. BFRT = blood flow restriction training.

BFRT involves applying pressure to restrict venous blood flow in the limbs, inducing localized muscle ischemia and hypoxia. This condition leads to the accumulation of metabolites, a decrease in pH, and an increase in metabolic stress,^[9,10]^ all of which stimulate muscle growth and improve function.^[11]^ While BFRT has been studied primarily in adults, its impact on adolescents, who are still in critical stages of physical development, may differ due to their unique hormonal and growth characteristics. This section aimed to review the mechanisms of BFRT, focusing on how these may apply to adolescents specifically (Fig. 1).

### 2.1. Metabolic stress and hormone response

BFRT is known to induce significant metabolic stress, which, in turn, triggers the release of growth hormone (GH)^[12]^ and insulin-like growth factor 1 (IGF-1),^[13]^ both crucial for muscle muscle development and bone growth in adolescents.^[14]^ Therefore, the metabolic stress induced by BFRT is particularly relevant for adolescents, as it can mimic the hormonal responses typical of high-load training but with reduced risk.

Although most research on GH and IGF-1 responses in BFRT focuses on adults, studies have demonstrated that increases in these hormones correlate with muscle cross-sectional area enhancement.^[15]^ In adolescents, this mechanism could similarly stimulate muscle hypertrophy and strength gains. Further research is warranted to confirm whether the hormonal responses to low-load BFRT in adolescents can effectively support healthy muscle and skeletal development.

### 2.2. Regulation of protein synthesis via the mTOR pathway

Protein synthesis regulation is crucial to BFRT-induced muscle adaptation, largely through the activation of the mechanistic target of rapamycin (mTOR) pathway, which promotes protein synthesis and inhibits protein breakdown.^[16]^ This pathway is influenced by key proteins like S6 kinase 1 and myostatin (MSTN), both involved in muscle growth.^[17,18]^ Under low-intensity conditions, BFRT increases phosphorylation of SK61, thereby enhancing protein synthesis.^[19]^ For adolescents, whose musculoskeletal systems are still developing, this low-intensity BFRT mechanism offers an effective means of supporting muscle growth without imposing the higher mechanical load typically associated with conventional resistance training, thus presenting a low-risk, high-benefit training modality. In addition, MSTN acts as a negative regulator of skeletal muscle growth, primarily by inhibiting the proliferation of myoblasts.^[20]^ BFRT has been shown to reduce MSTN signaling, further promoting muscle hypertrophy. Consequently, the mTOR signaling is particularly relevant in adolescents, whose skeletal muscle growth depends on sustained protein synthesis to support ongoing development.

### 2.3. Muscle fiber recruitment and neural adaptation

Under normal circumstances, muscle contraction first recruits slow-twitch muscle fibers (type I) and subsequently fast-twitch muscle fibers (type II). BFRT, by inducing hypoxia and metabolite accumulation, activates group III/IV afferent neurons, accelerating the recruitment of type II fibers even under low-load conditions.^[21]^ This neural adaptation is particularly advantageous for adolescents, as high-load training may increase their risk of musculoskeletal injuries due to their developing musculoskeletal systems.^[22]^

In adolescents, low-load BFRT can thus achieve type II muscle fiber recruitment comparable to that seen in high-load training while minimizing injury risk.^[23]^ This higher neural activity at low-intensity supports efficient muscle hypertrophy in adolescents, offering both safety and effectiveness for this age group.

### 2.4. Cell swelling and anabolic response

The venous blood flow restriction induced by BFRT may also cause cellular swelling response due to an increased osmotic gradient from metabolite accumulation, resulting in fluid influx into muscle cells.^[24]^ This cell swelling activates the mTOR signaling pathway, promoting an anabolic response and inhibiting catabolism.^[19]^

For adolescents, the cell swelling response could trigger metabolic adaptations similar to those produced by high-load training, potentially increasing muscle cross-sectional area. However, it is essential to consider the developmental stage of each individual, as the cellular and tissue responses to mechanical stress in adolescents may differ from those in adults. Future studies should explore the specific effects of cell swelling in adolescent BFRT to ensure both its safety and efficacy.

## 3. Effects of BFRT on adolescent muscle

The adolescents period, defined by the World Health Organization as ages 10 to 19, is crucial for physical and mental development during which training can positively or negatively impact adolescents’ growth depending on training intensity and type.^[25]^ Adolescence is often categorized into early (10–14 years) and late (15–19 years) stages, each associated with distinct hormonal profiles, musculoskeletal maturation, and neuromuscular coordination. These differences may influence how adolescents respond to various training interventions. It is essential that exercise protocols respect developmental stages to avoid injury or overtraining. BFRT is particularly promising for adolescents as it offers muscle hypertrophy, strength, and endurance improvements at low loads, reducing the risk of strain and injury. By combining low load, short duration, and high efficiency, BFRT may provide a safer alternative to traditional resistance training in promoting adolescent muscle fitness.

### 3.1. Muscle hypertrophy

Muscle hypertrophy, or the increase in muscle cross-sectional area, depends on muscle fiber number and thickness. BFRT has been shown to significantly increase the circumference and cross-sectional area of upper arm and thigh.^[26]^ Similar effects of BFRT observed in youth athletes, demonstrating its applicability to adolescent muscle development.^[27]^ For instance, a study conducted on college students showed a 10% increase in rectus femoris and medialis muscle thickness after 12 weeks of BFRT.^[28]^ However, other studies suggest that BFRT needs to be conducted for more than 8 weeks to induce noticeable muscle adaptations, which should be considered when designing adolescent training programs.^[29]^

### 3.2. Muscle strength

Muscle strength, typically measured by maximum force output, can also be enhanced with BFRT even at low intensities. The American College of Sports Medicine recommends a load of 60% to 70% of 1-repetition maximum for strength development.^[30]^ However, BFRT can effectively improve muscle strength at intensities below 40% 1-repetition maximum,^[31]^ which is crucial for adolescents, whose developing skeletal and muscular systems are more susceptible to damage from high-intensity loads. Studies analyzing the effects of BFRT on various muscle groups, including the elbow flexion, knee extension, and core muscles, found that BFRT significantly improves overall strength, even in low-load conditions.^[26]^ These findings support the use of BFRT as a supplemental to traditional resistance training for adolescents, offering strength improvements without exposing them to higher mechanical loads and potential injury.

### 3.3. Muscular endurance

Muscular endurance refers to the ability to sustain muscle activity over time. BFRT has been shown to enhance endurance by increasing muscle hypertrophy and strength.^[32]^ For example, studies combining BFRT with rotator cuff exercises has found superior improvements in shoulder and arm endurance and mass compared to conventional training.^[33]^ In youth athletes, such as football players, BFRT incorporated into sport-specific conditioning programs has been associated with improved endurance and reduced training volume.^[34]^

However, some studies have reported limited effects of BFRT on endurance during the warm-up phases, highlighting the need for further research to optimize BFRT for endurance training in adolescents.^[35,36]^ Future studies should systematically evaluate how training frequency, intensity, and duration of BFRT affect endurance, particularly in adolescent athletes who may benefit from BFRT’s lower mechanical load yet high adaptation potential.

### 3.4. Muscle activation and fatigue

BFRT training promotes neuromuscular adaptations by increasing muscle activation at low intensities.^[37]^ This is particularly beneficial for adolescents, as high-intensity training could pose a risk to their developing musculoskeletal systems. Low-intensity BFRT increases activation of key muscles, such as the triceps and anterior deltoid, while also eliciting higher subjective fatigue due to the increased neural engagement and metabolic stress associated with blood flow restriction.^[38,39]^ Studies have demonstrated that BFRT-induced muscle activation benefits both restricted and unrestricted muscle areas, leading to functional performance gains across multiple muscle groups.^[40–42]^

Recent research on high-level athletes has shown that BFRT, particularly with compression settings around 50% of arterial occlusion pressure (AOP), optimizes muscle activation while reducing mechanical strain on joints, making it a practical option for adolescent training and injury prevention.^[43]^ These findings support BFRT as a tool for enhancing muscle activation in adolescents, promoting performance and functional gains without excessive loading.

While current evidence on BFRT in adolescents rarely addresses sex-specific responses, it is important to consider potential physiological differences between male and female adolescents – such as differences in hormonal fluctuations, muscle mass distribution, and recovery patterns – which may influence training adaptations. Future research should explore these sex-based differences to further individualize and optimize BFRT protocols for both boys and girls.

## 4. BFRT implementation pathway for adolescent

Given the unique physiological characteristics of adolescents, an individualized BFRT protocol is essential to ensure both effectiveness and safety. This implementation pathway for BFRT in adolescents can be divided into 3 main categories: physical health improvement, athletic performance enhancement, and sports injury rehabilitation. Table 1 provides individualized recommendations based on AOP for different adolescent populations to optimize training benefits.

**Table 1:** Individualized BFRT program recommendations for adolescents based on AOP.

Target population	Load (% 1RM)	Pressure (% AOP)	Pressurized site	Frequency/duration	Training program
Healthy college students	20	40%–60%	Proximal thigh	3 times/wk; 4 sets/time; 12 wk	Half squat
Obesity	20	40%	Brachial/femoral artery	3 times/wk; 4 sets/time; 18 wk	Half squat; Lateral raise
Upper-body training	40	40%–50%	Proximal upper arm	3 times/wk; 30 min/time; 8 wk	Flat bench press; Barbell pull-up
Lower-body training	20	50%	Upper 1/3 of thigh	3 times/wk; 20–30 min/session; 6 wk	Vertical jump; Half squat; Sprint running
Knee injury rehabilitation	30	50%–70%	Proximal thigh	3 times/wk; 8 wk	Knee flexion/extension; Straight leg raise; Quadriceps isometric contraction
Ankle injury rehabilitation	25–50	50%	Above the knee	3 times/wk; 6 wk	Ankle valgus; Dorsiflexion training

1RM = 1-repetition maximum, AOP = arterial occlusion pressure, BFRT = blood flow restriction training.

### 4.1. Applications for enhancing physical health

Safeguarding optimal physical health is critical during adolescence and includes key fitness components like endurance, strength, and flexibility. Studies have shown that BFRT, when combined with low-load exercise, significantly enhances skeletal muscle mass and improves body composition.^[44,45]^ For example, a randomized controlled trial among obese college students found that combining BFRT with cycling (set at 40% maximum oxygen uptake) improved indicators such as body fat percentage, blood lipids, and other serum markers, which demonstrates the value of BFRT for managing health and fitness in adolescents, particularly in post-pandemic settings where physical inactivity has been an issue.^[46]^

For adolescents aiming to reduce body fat, BFRT targeting key areas like the brachial and femoral arteries at intensities around 40% of AOP, combined with exercises such as 20% 1RM half squat and lateral hip abduction raises, may be beneficial. Conversely, for healthy adolescents seeking to enhance muscle strength and endurance, the AOP percentage may be gradually increased to 50% to 60%. This gradual adjustment allows flexibility in BFRT protocols to accommodate different fitness goals among adolescents.

### 4.2. Applications for enhancing athletic performance

BFRT has shown promising results in improving specific performance attributes critical to athletic success.^[47]^ Evidence indicates that BFRT supports muscle hypertrophy and function, contributing to enhanced athletic performance. For instance, Yasuda et al have found that long-term BFRT – defined as a 12-week intervention – led to substantial increases in muscle cross-sectional area and strength without diminished effects over time or load increases.^[47–49]^ In recent years, other studies further support BFRT’s efficacy in enhancing key performance indicators, such as sprint speed, vertical jump height, and agility, which are crucial for sports requiring explosive power and endurance like tennis and handball.^[8,35,50]^

To maximize BFRT’s effectiveness for adolescent athletes, specific compression settings are recommended based on sport type. For upper-body sports (such as badminton, table tennis, and volleyball), BFRT with 40% to 50% AOP has been effective in increasing strength and endurance in target muscles. For lower-body sports (such as football and sprinting), a 50% AOP setting on the upper thigh has been shown to improve explosive strength, agility, and overall performance. These sport-specific BFRT applications offer a versatile, lower-risk alternative to high-load resistance training, which can pose injury risks to the developing musculoskeletal system of adolescents.

### 4.3. Applications for sports injury rehabilitation

Adolescents recovering from sports injuries often face challenges such as muscle weakness and atrophy, especially in musculoskeletal injuries. BFRT offers a viable method for rehabilitation by enhancing muscle strength without excessive load on healing tissues. Clinical studies have demonstrated that low-load BFRT effectively restores quadriceps strength and endurance after anterior cruciate ligament reconstruction, while also significantly alleviating pain and reducing joint effusion.^[51,52]^ Hughes et al^[51]^ also reported that BFRT provided greater pain relief and muscle adaptation compared to traditional methods, highlighting its utility for both injured and recovering patients.

Moreover, BFRT has been applied effectively in cases of chronic ankle injury, tendinopathy, and shoulder injuries, supporting muscle adaptation during rehabilitation and facilitating safe, efficient recovery.^[53,54]^ For adolescent rehabilitation, BFRT with 50% to 70% of AOP can effectively prevent joint contracture, maintain muscle function, and support a safe return to physical activity. With its low-risk profile, BFRT offers a tailored approach for adolescent rehabilitation, helping young athletes maintain and regain strength and function without straining healing tissues.

## 5. Implications for research

BFRT’s mechanisms in adolescents are not yet fully understood, especially concerning its impact on hormonal responses and muscle growth. This study aims to advance the theoretical understanding of BFRT in adolescent populations, setting the stage for future studies that examine BFRT-specific adaptations in this age group, with special attention to safety and long-term developmental outcomes.

Extensive research has confirmed the adaptive effect of BFRT on muscle hypertrophy and strength growth across various populations. The primary physiological mechanisms underlying BFRT are driven by metabolic stress and mechanical tension, which promote the secretion of anabolic hormones such as GH, IGF-1, and MSTN levels, thereby facilitating muscle growth.

Using BFRT at low load intensities (20%–40% of 1RM) has demonstrated a low risk of injury, making it particularly suitable for adolescents. By applying individualized pressures based on AOP, BFRT ensures safety while effectively maintaining or improving muscle size, strength, and endurance. The lower load requirements reduce delayed-onset muscle soreness and help adolescents maintain a sustainable exercise routine. This study provides a theoretical basis for BFRT and lays a foundation for further scientific, targeted guidance in youth strength training.

Future research should focus on optimizing BFRT protocols specific to adolescent physiology, including ideal AOP percentages, load intensities, and training frequencies for different growth stages. Additionally, long-term studies are needed to assess the impact of BFRT on adolescent musculoskeletal development and overall health outcomes.

## 6. Conclusion

In conclusion, BFRT offers significant potential in enhancing physical health, preventing sports injuries, and improving athletic performance in adolescents. Nevertheless, given the ongoing maturation and ossification of epiphyseal cartilage during this developmental stage, high-intensity strength training may increase the risk of musculoskeletal injuries and could negatively impact development. In contrast, BFRT provides an effective, low-risk alternative, allowing adolescents to achieve similar muscle adaptations to high-intensity training with reduced mechanical strain.

As an ongoing developing training method that enriches physical education and sports training practices, BFRT supports the development of basic physical fitness and sports-specific performance. With appropriate AOP-based pressure settings, BFRT can be a valuable tool for promoting adolescent health and physical fitness. Therefore, BFRT is recommended as an innovative approach for schools, parents, and sports organizations to incorporate into youth fitness programs to foster safe and effective physical development in adolescents.

## Acknowledgments

The authors would like to thank Humanities and social science research Foundation of Ministry of Education of China for their support in publishing this article.

## Author contributions

**Conceptualization:** Zhen-Lei Chen, Tian-Shu Zhao.

**Data curation:** Zhen-Lei Chen, Tian-Shu Zhao.

**Formal analysis:** Zhen-Lei Chen, Tian-Shu Zhao, You-Qing Shen, Li-Ping Huang.

**Funding acquisition:** Li-Ping Huang.

**Investigation:** You-Qing Shen.

**Methodology:** You-Qing Shen.

**Resources:** Si-Zhuo Zhang.

**Software:** Shuang-Feng Ren, Si-Zhuo Zhang.

**Supervision:** Shuang-Feng Ren, Si-Zhuo Zhang, Ji-Lai Xu.

**Validation:** Shuang-Feng Ren, Ji-Lai Xu.

**Visualization:** Zhen-Lei Chen, Tian-Shu Zhao, Ji-Lai Xu.

**Writing – original draft:** Zhen-Lei Chen, Tian-Shu Zhao, Li-Ping Huang.

**Writing – review & editing:** Zhen-Lei Chen, Tian-Shu Zhao, Li-Ping Huang.
